# Supplementary material for: Switch-like and persistent memory formation in individual Drosophila larvae
Source: eLife. 2021 Oct 12;10:e70317. doi: 10.7554/eLife.70317 (PMC8510578; doi:10.7554/eLife.70317)
Supplement: Supplementary file 1. [file elife-70317-supp1.zip › Schematics/lesar_elife_schematics.pdf]

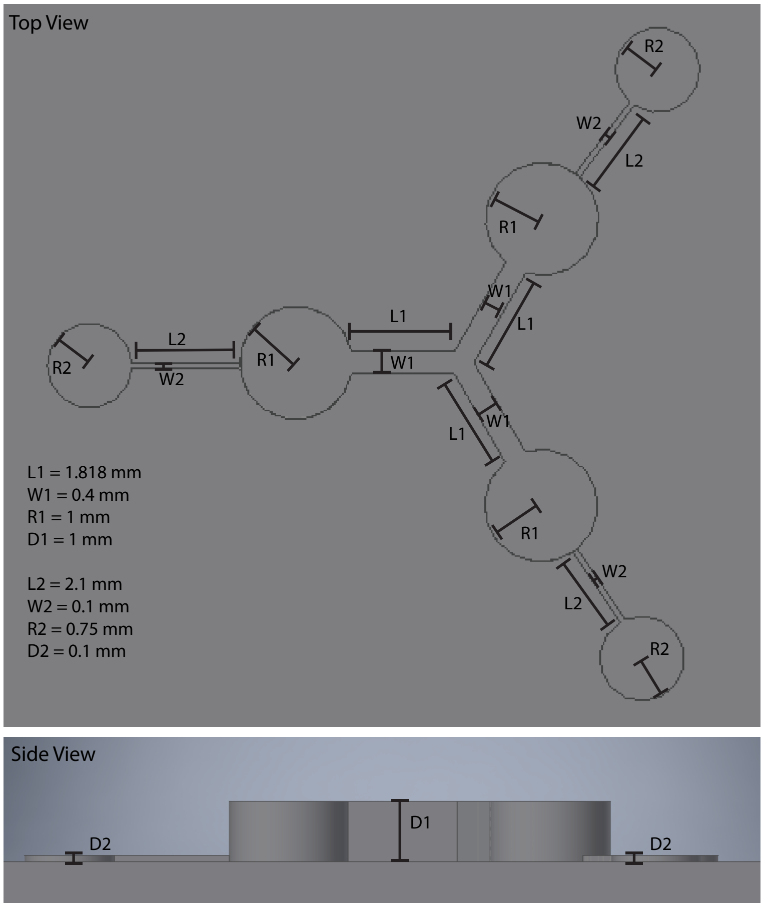

Figure 1: **Y-maze schematic.** Top and side view of Y-maze assay. The dimensions of the Y-maze are included on the schematic. The Y-mazes were designed in Autodesk Inventor (see ymaze.ipt) and 3D printed on an Ember 3D printer using black prototyping resin. 4% agarose was poured over the masters and allowed to solidify; then mazes were removed from the mold. Agarose Y-mazes were stored in tap water before use. The masters could be reused.

The CAD file for the Y-maze is **ymaze.ipt**, and was designed with the program Autodesk Inventor.

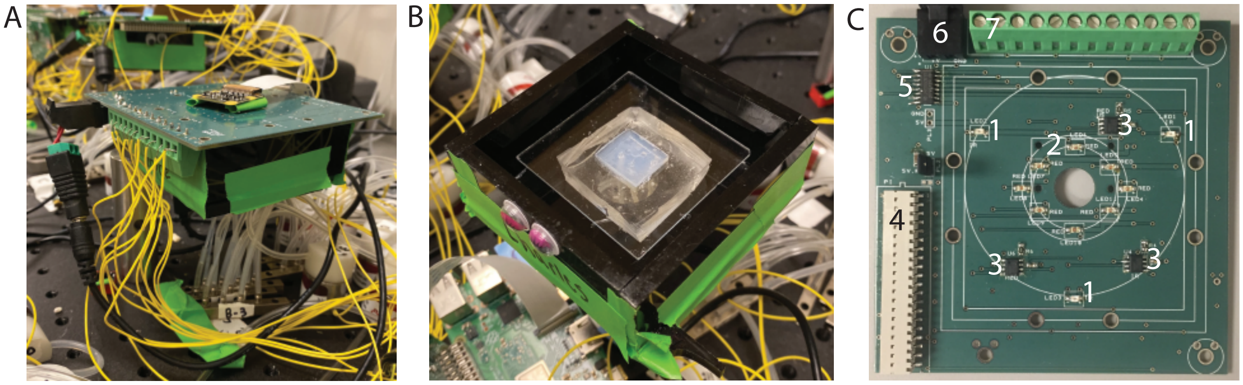

Figure 2: **Our experimental setup.** (A) The black box which houses each Y-maze. The board on top is connected to a Raspberry Pi, and has IR LEDs (for camera illumination) and red LEDs (for reward presentation during training), as well as hookups for the valves. The camera can be seen on top. Eight copies of this setup were built. (B) An agarose Y-maze in the setup. Each Y-maze sits inside a PDMS base; tubing is secured in the PDMS base and connects to each maze. (C) Our custom circuit, which controls the direction of airflow and LEDs. 1) IR LEDs; 2) Red LEDs; 3) LED Drivers (one driver controls three IR LEDs; two drivers each control four red LEDs); 4) Raspberry Pi GPIO header; 5) Valve driver; 6) Valve power source; 7) Valve connection header (six valves; one CO_2 valve and one vacuum valve for each of the three channels in the Y-maze). See Figure 5 for circuit schematic details.

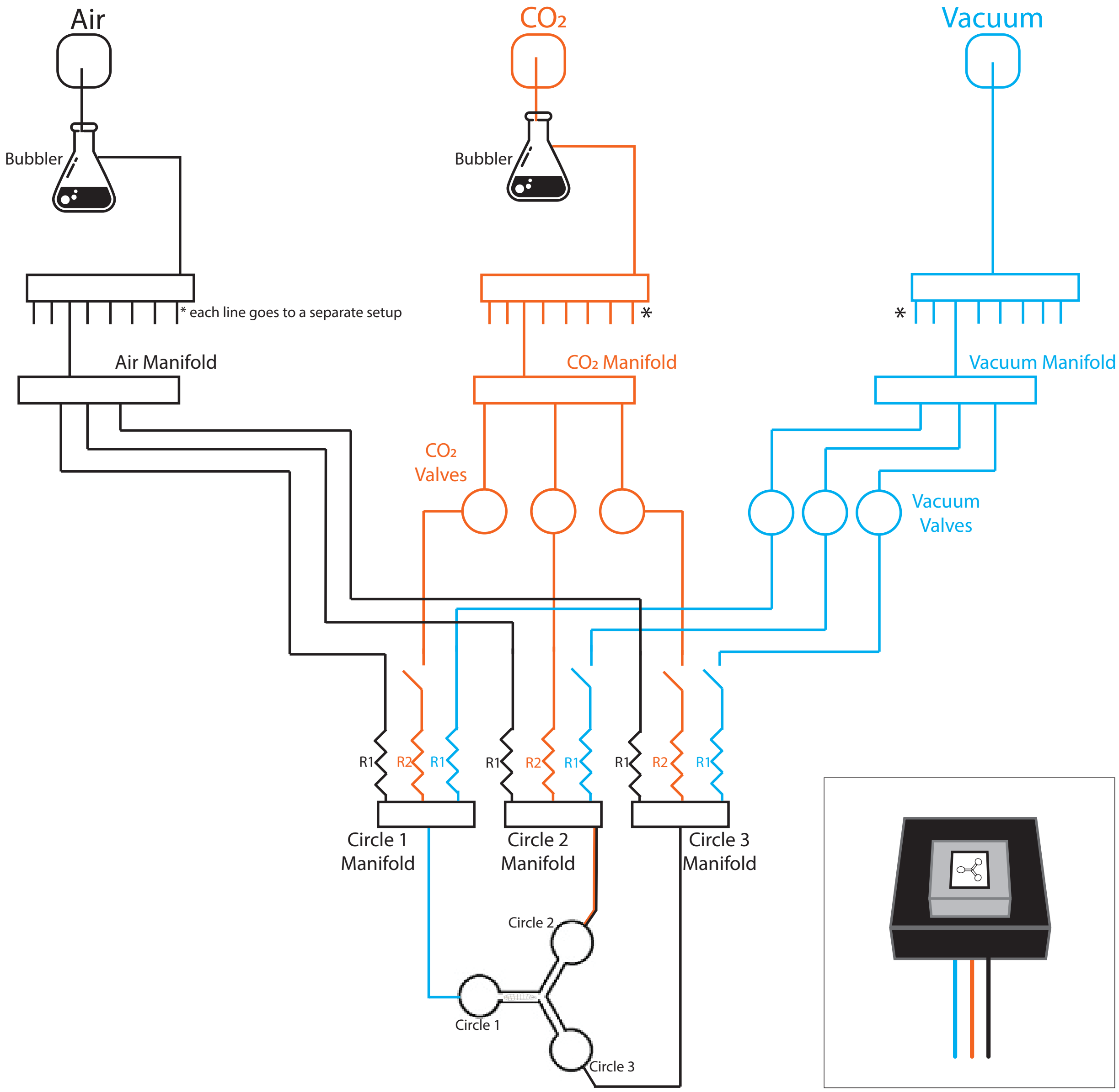

Figure 3: **Tubing network for our behavioral setup.** Pressure for air (black), CO_2 (orange), and vacuum (blue) were controlled at the sources. Our sources for air and CO_2 are humidified through a bubble humidifier. Then, air, CO_2, and vacuum tubing to individual assays were separated through a block manifold. We show one representative individual assay in the schematic; each of the eight setups were identical and indicated at *. After being separated towards an individual assay, the air, CO_2, and vacuum is separated by another manifold for the three circles. Air is flowing constantly. CO_2 and vacuum tubing go through solenoid pinch valves, controlled by a custom circuit we designed. Each channel has either air, a combination of air and CO_2, or vacuum. The CO_2 concentration in the odorized channels was controlled by a resistive network of tubing connected to the air and odor sources. Tubing with different inner diameters and/or lengths was used as a way to control the flow (noted as resistors R1 and R2). In this schematic, we show an example where the outlet (vacuum) is circle 1; circle 2 is the randomly chosen CO_2 channel containing a mix of air and CO_2; and circle 3 contains pure air. Insert: view from above of a Y-maze in PDMS base (gray), placed inside acrylic box (black), with tubing underneath. R1: 0.020" ID, 1" long. R2: 0.010" ID, 2" long.

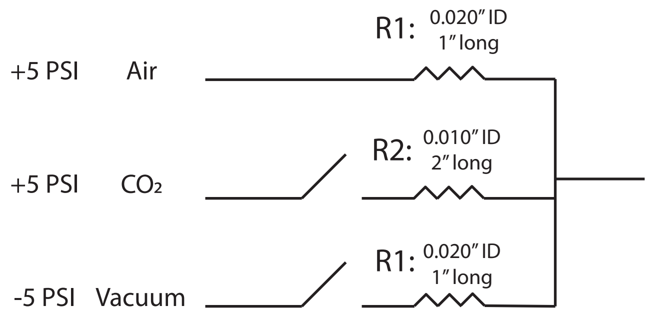

Figure 4: **Resistive tubing network.** Schematics for the resistive tubing network. The CO_2 concentration in the odorized channels was controlled by a resistive network of tubing connected to the air and odor sources. Resistance was controlled using tubing of different lengths and inner diameters. For CO_2 and vacuum, resistors came after valves. This inexpensive alternative to a mass-flow controller produced a stable ratio of odor to air that was consistent from day to day and independent of the overall flow rate. See Figure 3 for the tubing setup.

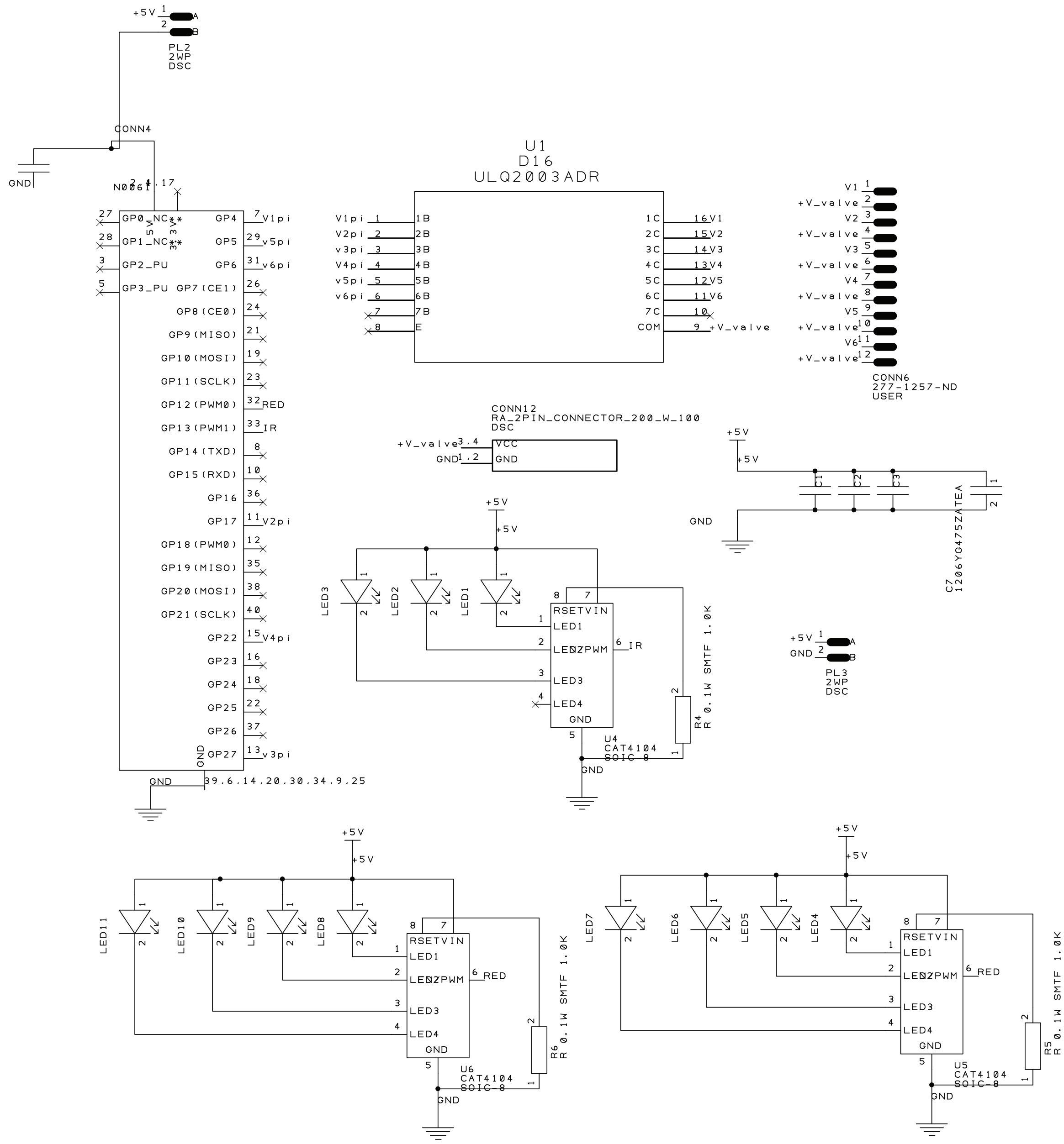

Figure 5: **PCB Schematic.** Our custom circuit, which controls the direction of airflow and the LEDs. The CO_2 concentration in the odorized channels was controlled by a resistive network of tubing connected to the air and odor sources; the direction of flow was controlled by solenoid pinch valves, actuated by a custom circuit we designed. The circuit contains a driver for the 6 valves, a header for the valves to the board, and a power source for the valves (+12 V). The circuit includes 3 IR LEDs for camera illumination, 8 red LEDs for reward presentation during training, and 3 LED Drivers (one driver controls three IR LEDs; two drivers each control four red LEDs). The circuit also includes a Raspberry Pi GPIO header.

The schematics for the circuit can be found in the file **lightring.sch**; the schematic was designed using the program PCB Artist. We then translated the schematic into a PCB design using the PCB Artist PCB design layout editor. The PCB file can be found at **lightring.pcb**. The PCB board has two layers. See Figure 2C for PCB board.
